# TSH Pulses Finely Tune Thyroid Hormone Release and TSH Receptor Transduction

**DOI:** 10.1210/endocr/bqad164

**Published:** 2023-11-02

**Authors:** Anne Guillou, Yasmine Kemkem, Chrystel Lafont, Pierre Fontanaud, Davide Calebiro, Pauline Campos, Xavier Bonnefont, Tatiana Fiordelisio-Coll, Ying Wang, Emilie Brûlé, Daniel J Bernard, Paul Le Tissier, Frederik Steyn, Patrice Mollard

**Affiliations:** Institute of Functional Genomics, University of Montpellier, CNRS, INSERM, Montpellier 34094, France; Institute of Functional Genomics, University of Montpellier, CNRS, INSERM, Montpellier 34094, France; Institute of Functional Genomics, University of Montpellier, CNRS, INSERM, Montpellier 34094, France; Institute of Functional Genomics, University of Montpellier, CNRS, INSERM, Montpellier 34094, France; Institute of Metabolism and System Research (IMSR), University of Birmingham, Birmingham B15 2TQ, UK; Centre of Membrane Proteins and Receptors (COMPARE), Universities of Nottingham and Birmingham, Birmingham B15 2TQ, UK; Institute of Pharmacology and Toxicology, University of Würzburg, Würzburg 97078, Germany; Institute of Functional Genomics, University of Montpellier, CNRS, INSERM, Montpellier 34094, France; College of Engineering, Mathematics and Physical Sciences, University of Exeter, Exeter EX4 4SA, UK; Institute of Functional Genomics, University of Montpellier, CNRS, INSERM, Montpellier 34094, France; Laboratorio de Neuroendocrinología Comparada, Departamento de Ecología y Recursos Naturales, Biología, Facultad de Ciencias, Universidad Nacional Autónoma de México, Ciudad Universitaria, 04510 México, DF, México; Department of Pharmacology and Therapeutics, McGill University, Montreal H3G 1Y6, Canada; Department of Anatomy and Cell Biology, McGill University, Montreal H3G 1Y6, Canada; Department of Pharmacology and Therapeutics, McGill University, Montreal H3G 1Y6, Canada; Department of Anatomy and Cell Biology, McGill University, Montreal H3G 1Y6, Canada; Integrated Program in Neuroscience, McGill University, Montreal H3G 1Y6, Canada; Centre for Discovery Brain Sciences, University of Edinburgh, Edinburgh EH8 9XD, UK; School of Biomedical Sciences, The University of Queensland, Brisbane, Queensland 4072, Australia; Institute of Functional Genomics, University of Montpellier, CNRS, INSERM, Montpellier 34094, France

**Keywords:** ELISA, TSH, mouse models, cAMP regulation, thyroid, hypothyroidism

## Abstract

Detection of circulating TSH is a first-line test of thyroid dysfunction, a major health problem (affecting about 5% of the population) that, if untreated, can lead to a significant deterioration of quality of life and adverse effects on multiple organ systems. Human TSH levels display both pulsatile and (nonpulsatile) basal TSH secretion patterns; however, the importance of these in regulating thyroid function and their decoding by the thyroid is unknown. Here, we developed a novel ultra-sensitive ELISA that allows precise detection of TSH secretion patterns with minute resolution in mouse models of health and disease. We characterized the patterns of ultradian TSH pulses in healthy, freely behaving mice over the day-night cycle. Challenge of the thyroid axis with primary hypothyroidism because of iodine deficiency, a major cause of thyroid dysfunction worldwide, results in alterations of TSH pulsatility. Induction in mouse models of sequential TSH pulses that mimic ultradian TSH profiles in periods of minutes were more efficient than sustained rises in basal TSH levels at increasing both thyroid follicle cAMP levels, as monitored with a genetically encoded cAMP sensor, and circulating thyroid hormone. Hence, this mouse TSH assay provides a powerful tool to decipher how ultradian TSH pulses encode thyroid outcomes and to uncover hidden parameters in the TSH-thyroid hormone set-point in health and disease.

Pulsatile intercellular communication is a critical mode of information transfer in the animal kingdom (1). In mammals, hormone pulsatility is essential for homeostasis and, in general, pulsatile hormone patterns are more efficient that continuous hormone delivery in controlling tissue target function (2). Understanding the outcome of patterned hormone output has been seminal for translational applications, as was shown for the design of recombinant GH injection protocols in GH-deficient children (3).

Despite its importance, the role of pulsatility remains unknown for some key hormones controlling homeostasis and metabolism such as the TSH (4), which is secreted in a basal (nonpulsatile) and pulsatile manner by the human anterior pituitary gland (5, 6). Heterodimeric TSH synthesis and release is critical for thyroid hormone (TH) synthesis and secretion (5, 6) and is regulated by hypothalamic-thyrotropin-releasing hormone (TRH) input and negative feedback exerted by triiodothyronine (T3) and thyroxine (T4) (7-9). Because small variations of T4 associated with disease can lead to large, detectable changes in TSH levels, monitoring circulating TSH is routinely used as a major index of thyroid function assessment (10). Remarkably, this diagnostic tool is only based on a single TSH time point because the relevance of patterned TSH secretion is unknown. However, the use of a single time point detection of circulating TSH as a reference is widely debated (11, 12), specifically in defining the TSH-TH set-point that best reflects thyroid status in humans.

This lack of understanding has prompted us to establish an experimental framework in mice to examine the dynamics and role of ultradian TSH pulse patterns both on TSH receptor downstream signalling in isolated thyroid follicles, and on the release of thyroid hormones in vivo. Using a novel ultra-sensitive ELISA to overcome the lack of available tools sensitive enough to resolve minute-range TSH fluctuations in rodents (13, 14), we characterized TSH pulsatility in mice. We then explored alteration of ultradian TSH pulses in hypothyroidism by monitoring both TSH secretion profiles and thyroid hormone levels on the establishment of primary hypothyroidism resulting from iodine deficiency, a leading cause of thyroid dysfunction worldwide (15, 16). Finally, we compared the efficiency of a series of short-lived TSH pulses vs prolonged changes in TSH levels at stimulating murine thyroid follicles using genetically modified mice expressing a fluorescent cAMP sensor as a proxy for TSH-receptor downstream signalling (17) and monitoring increased TH levels in vivo. This framework revealed the critical importance of TSH pulsatility in periods of minutes in the control of thyroid signal-transduction and hormone levels in mice, providing a model system for TSH role in thyroid axis regulation in health and disease.

## Materials and Methods

### Animals

Wild-type FVB/NJ mice were purchased from Janvier-SAS (Le Genest-St-Isle, France). Mice were given chow and water ad libitum and were housed in a conventional facility on a 12-hour light/12-hour dark cycle; the time of lights on was defined as zeitgeber time = 0. Animals were housed for 15 days in an inverted 12-hour dark/12-hour light cycle for “dark phase” blood sampling. Hypothyroidism was induced with an iodine-deficient diet (low-iodine diet [LID]) enriched with 0.15% propylthiouracil (LID + 0.15% propylthiouracil diet TD.95125; Envigo, USA). Experiments were conducted according to the European guidelines for animal welfare (2010/63/EU). Protocols were approved by the Institutional Animal Care and Use Committee (CEEA-LR-1434) and the French Ministry of Agriculture (APAFIS#21598). Wild-type or transgenic FVB mice expressing the Epac1-camps sensor under control of the cytomegalovirus enhancer/chicken β-actin promoter (17) were used for the isolation of primary thyroid cells. All animal work was done according to regulations of the relevant authority, the government of Lower Franconia, Bavaria.

### Anesthesia, Catheterization and Injections

Mice were anesthetized by inhalation of isoflurane (1.5% in O_2_). Animal temperature was maintained at 36 °C (heating pad; FHC). A catheter was inserted into the jugular vein for blood collection and drug administration. A total 50 µL of blood was collected at the beginning and end of each experiment for total T4 measurement. Tail-tip blood collection was performed concomitantly for mouse thyroid-stimulating hormone (mTSH) measurements. In anesthetized mice, mTSH (AFP9090D, NIDDK-NHPP) was injected at a dose of 8 ng/50 µL. mTSH was either acutely injected or perfused using a pump at a rate of 10 µL/minute for 10 minutes. For mTSH half-life measurement in restricted awake animals, mTSH was injected at a dose of 30 ng/50 µL, via the tail vein. TRH (P13-19, Sigma) was injected IP at a dose of 10 µg/kg.

### Tail-tip Whole Blood Sampling

Tail-tip blood sampling in freely behaving animals was performed as described previously (18). Briefly, 1.5 µL or 3 µL of blood droplets were collected at the tail tip and immediately diluted (1:20) in PBS-T (PBS, 0.05% Tween-20), and promptly frozen at −20 °C until use within a month or otherwise stored at −80 °C.

### mTSH Sandwich ELISA

**Figure 1.**
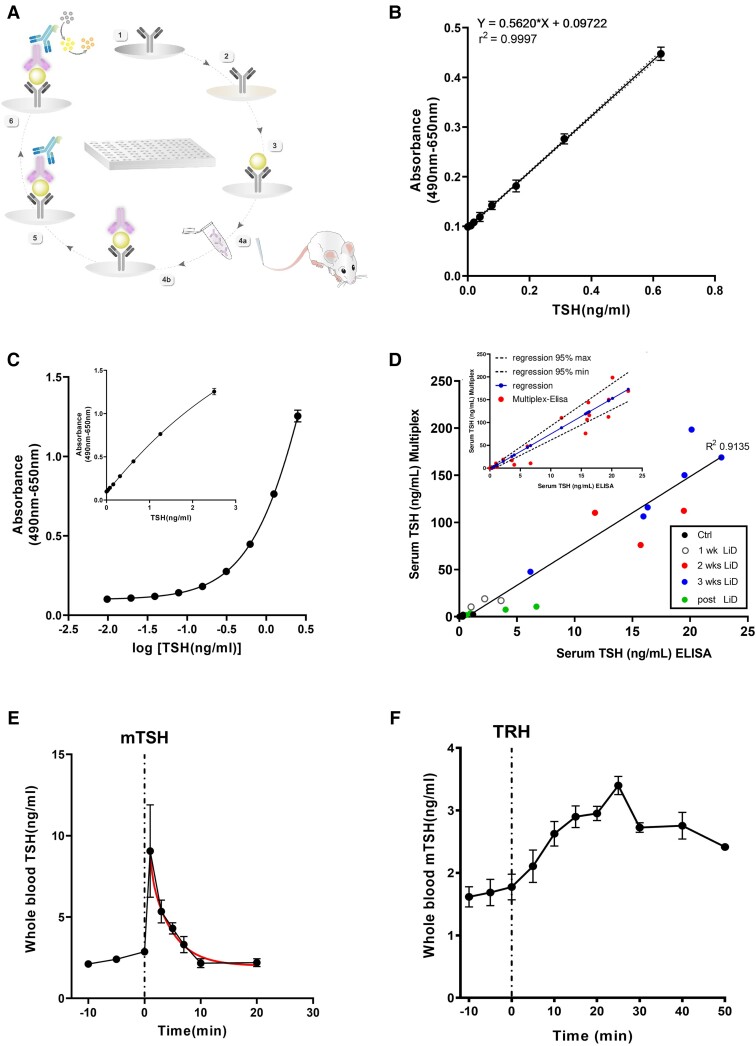
Development of an ultra-sensitive mouse TSH ELISA. (A) Schematic representation of the ultra-sensitive mouse TSH ELISA. The ELISA plate is coated with mouse TSH hormone antiserum (guinea pig) (A1); a blocking buffer is added to reduce nonspecific binding (A2); 1.5 µL or 3 µL of mouse whole blood tail-tip samples diluted (1:20) in PBS-T (PBS, 0.05% Tween-20) are then loaded (A3); detecting anti-rat beta TSH antibody (rabbit) is blocked (A4a) and incubated (A4b); secondary horseradish peroxidase (HRP)-conjugated anti-rabbit antibody is added (A5); finally, a substrate results in an enzymatic colorimetric reaction. (B) Mean linear standard curve of repeated analysis of mTSH standards ranging from 0.0097 to 0.625 ng/mL (n = 11). mTSH standard values are plotted on the y axis; absorbance on the x axis. Linearity estimated with an *R*² value of 0.9997. (C) Nonlinear regression analysis of standards from 0.0097 to 2.5 ng/mL presents an *R*² value of 0.9813 or 0.9819, with a nonlinear Syx of 0.05244. (Inset) Same data plotted on a linear concentration scale. (D) Correlation of mTSH values from serum samples were obtained using Multiplex and ELISA assays. Serum samples were collected from freely behaving mice on hypothyroidism induction and LT4 treatment (14), with 1:10 dilution before assay with mTSH ELISA. (Inset) Passing and Bablok regression analysis illustrating the linear relationship between ELISA and Multiplex assays (*P* = .66894, cumsum adapted method; x and y scales as in Fig. 1D). (E) mTSH half-life was determined by 1-phase decay of the plotted mTSH profiles of 6 restrained awake animals injected with 30 ng mTSH via the tail vein. (F) IP TRH (10 µg/kg) injection induced an increase in mTSH secretion, which peaked 25 minutes after injection in freely behaving adult mice.

mTSH ELISA steps are shown in Fig. 1A. To assess mTSH level in 3-µL samples, a 96-well plate (Corning Costar assay plate #9018) was coated with 50 µL of capture antibody (mouse TSH antiserum—guinea pig, A.F. Parlow National Hormone and Peptide Program Cat# AFP98991, RRID:AB_2810234) at a final dilution of 1:15 000 in PBS (Na_2_HPO_4_ 7.6 mM; NaH_2_PO_4_ 2.7 mM; NaCl 0.15 M, pH 7.4). The plate was protected with parafilm and incubated in a humidified chamber overnight at 4 °C without shaking. The coating solution was removed and a specific washing step with 300 µL PBS per well was performed (3 minutes at room temperature [RT] on an orbital shaker [600 rpm]). Each well was then incubated with 200 µL blocking buffer (5% skim milk powder in PBS with 0.05% Tween-20 [PBS-T, 0.05%]) for 2 hours at RT under agitation (250 rpm) to reduce nonspecific binding. A standard curve was prepared using a 2-fold serial dilution of mouse TSH (mTSH reference preparation, AFP9090D, NIDDK-NHPP) in PBS-T supplemented with 0.2% bovine albumin serum. After a washing step of 300 µL PBS-T per well (3 minutes × 3 times at RT with shaking [600 rpm]), 50 µL of standards and samples were loaded and incubated for 2 hours at RT, under shaking. To reduce the cross-reaction with the coating antibody, the detection rabbit antiserum to rat beta TSH (A.F. Parlow National Hormone and Peptide Program Cat# AFP1274789, RRID:AB_2923323) was preincubated for 30 minutes at a final dilution of 1:5000 in blocking buffer with 5% (v/v) normal guinea pig serum before incubation. The plate was washed and loaded standards and samples were incubated with 50 µL of detection antibody solution for 90 minutes at RT with shaking. After a washing step, the complex was incubated with 50 µL horseradish peroxidase-conjugated antibody (antirabbit, IgG [H + L]; BE-A120-208P, Euromedex) at a final dilution of 1:3000 in 50% PBS/50% blocking buffer for 90 minutes at RT with shaking. Adding 100 µL O-phenylenediamine (1 mg/mL) (34006, Thermo Fisher Scientific) substrate per well in citrate phosphate buffer (citric acid 49 mM; Na_2_HPO_4_ 102 mM, pH 5.0) containing 0.03% hydrogen peroxide resulted in an enzymatic colorimetric reaction. The reaction was stopped after 30 minutes by adding 50 µL 2.5 M H_2_SO_4_ to each well. Absorbance was read at a wavelength of 490 nm with a correction at 650 nm using the TECAN Spark multimode microplate reader. The concentration of mTSH in each well was calculated by regression of the standard curve. For 1.5-µL samples, this protocol was tested and validated in a “Corning Costar assay plate half area ref: 3690” by dividing all the volumes by 2.

### Luminex Assay for mTSH

Serum TSH was measured with a Milliplex assay (detection range: 12.2-50 000 pg/mL) following the manufacturer's instructions (Millipore Cat# MPTMAG-49K, RRID:AB_2811194). The intra-assay coefficient of variation was 2.2% and the inter-assay coefficient of variation was 14.0% (14).

### TH Detection

Collected blood was left at RT for 30 to 45 minutes before centrifugation at 2500 rpm for 10 minutes at RT. Serum was stored at −20 °C until T4 measurement, using a T4 ELISA kit (DRG International Cat# EIA-1781, RRID:AB_2927536).

### Thyroid Follicle Isolation

Mouse thyroid follicles were isolated as described previously (17). Briefly, thyroid lobes were dissected from adult CAG-Epac1-camps transgenic mice. The lobes were collected in a 1.5-mL tube, containing 1 mL of digestion medium consisting of 100 U/mL collagenase I, 100 U/mL collagenase II, and 1 U/mL dispase, dissolved in DMEM/F-12. Enzymatic digestion was carried out for 1 hour in a 37 °C water bath, with manual shaking every 15 minutes. After digestion, isolated individual follicles were washed 3 times with culture medium and plated on glass-bottom, 35-mm Petri dishes, coated with a thin layer of collagen gel. The collagen gel was prepared by spreading 8 mL of a collagen solution (3 mg/mL in 0.2% acetic acid) onto the glass surface, followed by addition of a neutralizing solution (0.4 M NaHCO_3_,0.2 M HEPES [pH 7.4]). Follicles were maintained in DMEM/F-12 + 20% fetal calf serum (37 °C, 5% CO_2_).

### Real-time Monitoring of cAMP Levels in Intact Thyroid Follicles

Glass-bottom Petri dishes containing thyroid follicles isolated from CAG-Epac1-camps mice were placed on a Zeiss Axiovert 200 inverted microscope equipped with an oil-immersion 63 × objective, a polychrome IV light source (Till Photonics), a 505 DCXR beam splitter, and a Cool SNAP-HQCCD-camera (Visitron Systems). Forster resonance energy transfer (FRET) was monitored using MetaFluor 5.0 software (Molecular Devices) as the ratio between emission at 535 ± 20 nm (yellow fluorescent protein [YFP]) and emission at 480 ± 15 nm (cyan fluorescent protein [CFP]), upon excitation at 436 ± 10 nm. The imaging data were analyzed using MetaMorph 5.0 (Molecular Devices) and Prism (GraphPadSoftware) software, by correcting for spillover of CFP into the 535-nm channel and direct YFP excitation, to give corrected YFP/CFP ratio data. Images were acquired every 5 seconds, with 5 ms of illumination time. Photo bleaching was negligible during a 30-minute observation. Experiments were done at 37 °C. Thyroid follicles were kept under laminar-flow perfusion with a custom-built apparatus that allows for the rapid exchange between different extracellular solutions.

### Statistics

TSH secretion was quantified as mean ± standard error of the mean, area under the curve (AUC), area under the curve over baseline (AUC/min), coefficient of variation, and pulse frequency using GraphPad Prism 8.4.2 and custom-made Mat-Lab routines. Statistical significance was measured accordingly in each set of experiments. The threshold level for statistical significance was set at *P* < .05. Passing and Bablok regression analysis of both ELISA and Luminex data (Fig. 1D, inset) was performed using a Mat-Lab routine (19). Cumulative sum linearity test (cumsum, Kolmogorov-Smirnov adapted test) was performed to investigate possible significant deviation from linearity between both ELISA and Luminex data. A *P* value lower than 0.1 leads to a rejection of the null hypothesis that the relationship is nonlinear between both sets of data.

## Results

### Detection of TSH Secretion Fluctuations in Freely Behaving Mice

To detect TSH secretion fluctuations in mouse models at a resolution comparable to that of human studies (4, 20), we first developed and validated an ultra-sensitive sandwich ELISA using an approach similar to that previously described for other pituitary hormones (Fig. 1A; Table 1), which allows measurement in tail-tip blood microsamples (18). Combined with optimized blood collection techniques that markedly reduce sample size volumes, this assay allowed for longitudinal monitoring of blood hormone levels in the same individual mouse. TSH values reported below in the results section were corrected from dilutions in PBS during the ELISA assay and are expressed in ng/mL.

**Table 1.** Validation of the ultra-sensitive TSH assay and the tail-tip blood sample collection method

Repeatability precision	Mean ± SEM	CV (%)
Inter-assay CV (whole blood) (quality control)	6.970 ± 0.256	12.2
Intra-assay CV (whole blood +1.25 ng mTSH)	1.106 ± 0.015	3.286
Intra-assay CV (whole blood + 0.625 ng mTSH)	0.6126 ± 0.0136	5.434
Intra-assay CV (diluted whole blood 1:20)	0.657 ± 0.007	3.290
LOB/LOD/LOQ		
Blank (OD values)	0.09905 ± 0.00659 (n = 11)	
LOB (mean blank + 1.645* [SD blank])	LOB = 0.1350	
LOD = LOB + 1.645* (SD low concentration sample)	LOD = 0.1721 ng/mL	
Pipetting variability		
Whole blood sample 1	3.885 ± 0.038	3.133
Whole blood sample 2	6.629 ± 0.074	2.487
Accuracy/recovery		
2.5 ng/mL		82.9%
0.15625 ng/mL		86.473%
0.039 ng/mL		92.685%

mTSH recovery in whole blood samples was measured by supplementing samples with mTSH at concentrations ranging from 0.039 to 2.5 ng/mL. Intra-assay CVs were determined using whole blood samples with a known mTSH concentration of 1.25 ng/mL. The inter-assay CV, calculated from 11 independent measurements, was determined using samples from LID-treated animals. LOB (mean blank [OD values] + 1.645 × [SD blank]) and LoD (LOB + 1.645 × [SD low concentration sample] (0.00975 ng/mL)) were determined by extension of the low end of the standard curve. Pipetting variability was determined via repeat analysis of whole blood samples previously diluted 1:20.

Repeated analysis of TSH ranging from 0.0097 to 0.625 ng/mL showed standard dilution linearity (*R*² = 0.9997; [*n* = 11]) (Fig. 1B). A nonlinear regression analysis of standards allowed determination of murine TSH (mTSH) values within a larger range of 0.0097 to 2.5 ng/mL in 1.5-µL or 3-µL blood samples (Fig. 1C; *R*²= 0.9813, with a nonlinear Syx of 0.05244). Characteristic mTSH assay features, including spike recovery measurement (80%-90% accuracy for detecting mTSH changes within ng/mL ranges), repeatability, low-iodine diet (LoD) and blank, are summarized in Table 1. Recovery of blood samples ranging from 0.039 to 2.5 ng/mL was 83.14% on average. We measured an intra-assay coefficient of variation (CV) of 3.286% using whole blood samples with a known mTSH concentration of 1.25 ng/mL. The inter-assay CV, resulting from 11 independent experiments, was 12.2%. The limits of blank and LoD were 0.1350 (OD) and 0.1721 ng/mL, respectively. These results show that our ELISA assay allowed detection of murine TSH levels within a ng/mL range from blood microsamples in freely behaving mice. This ELISA detected mTSH changes on axis challenge that were comparable with hormone levels measured with commercial TSH multiplex assays (14). When testing this, we adapted our mTSH ELISA to use serum samples since commercial TSH multiplex assays were designed for sera but not blood samples. Dilution of serum samples collected from both euthyroid and hypothyroid mice showed a linear relationship between serum TSH measurements using our ELISA and those measured with the TSH multiplex assay (Fig. 1D, inset; analyzed using the Passing and Bablok method (21-23), *P* = .66894, cumsum adapted method). Both assays allowed detection of changes in TSH levels on induction of primary hypothyroidism and its treatment with LT4 (Fig. 1D, *R*² = 0.9135). Using the highly sensitive TSH ELISA, we verified detection of exogenous (Fig. 1E) and endogenous (Fig. 1F) mTSH. First, acute IV injection of mTSH resulted in a sharp increase in detectable mTSH followed by a rapid decay phase. Exponential analysis estimated a half-life of 2.7 minutes for mTSH (Fig. 1E, red curve). To measure endogenous mTSH secretion by pituitary thyrotrophs, we injected awake mice with TRH IP. The latter has been shown to exclusively stimulate *pars distalis*, but not *pars tuberalis* thyrotrophs (24). TRH triggered a 91.54% rise in detectable mTSH, which peaked 25 minutes after administration (Fig. 1F). Hence, our experimental framework allows detection of TSH fluctuations in periods of minutes in freely moving mouse models.

### Interrogation of the Plasticity of TSH Secretion Profiles in Mouse Models in Health and Disease

**Figure 2.**
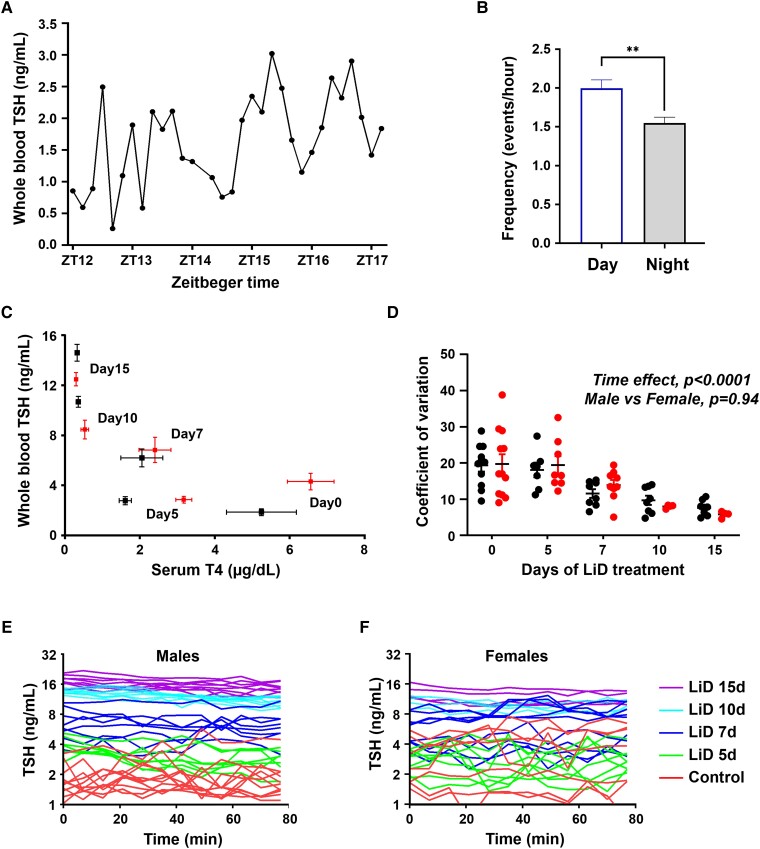
Ultradian TSH pulses in freely behaving euthyroid and hypothyroid mice. Awake mice were sampled every 10 to 15 minutes during 3- to 5-hour periods. (A) Representative example of ultradian mTSH secretion patterns detected in an adult male in the dark phase. (B) Analysis of TSH pulse frequency during the light vs night period (8 experiments). *P < .05. (C-D) Values measured in males and females are represented in black and red, respectively. (C) TSH/T4 relationship at days 0, 5, 7, 10 and 15 of low iodine + PTU 0.15% diet, in adult male and female mice. Numbers on graph (0, 5, 7, 10, and 15) correspond to the day of LID treatment. Means ± standard error of the mean are presented. (D) Distribution of coefficient of variation (CV) of mTSH secretion in male (black) and female (red) mice as a function of days 0, 5, 7, 10, and 15 of LiD treatment. (E-F) mTSH profiles (Log representation) in male (E) and female (F) mice at 0, 5, 7, 10, and 15 days of LID treatment.

We first applied our highly sensitive assay to characterize mTSH pulsatility in euthyroid freely moving mice (Fig. 2). Longitudinal 3- to 5-hour tail-tip blood microsampling with a frequency of 10 to 15 minutes during the diurnal and nocturnal period of rodent TSH secretion (25) detected ultradian mTSH pulses during both light and dark phases in individual adult male mice (Fig. 2A). We observed an increase in fluctuation frequency (**P = .0023; Mann-Whitney test, n = 8) in blood collections in the light phase compared with the early period of the dark phase (Fig. 2B). A significant decrease in TSH secretion over baseline (AUC) was measured between morning (Am) and afternoon (Pm) blood sample collections (***P = .0002; Mann-Whitney test, n = 8). Thus, the kinetics of TSH pulses in periods of minutes can be resolved over several hours in freely behaving mice with a resolution comparable to those reported in studies in humans (4).

We next applied our experimental approach to determine TSH secretion profiles at precisely controlled time points following alteration of the negative feedback exerted by thyroid hormones. For this, we applied a protocol to trigger primary hypothyroidism resulting from iodine deficiency in freely behaving mice (14). Male and female mice were both fed with an iodine-deficient diet enriched with propylthiouracil (LID) and mTSH secretion profiles were monitored over a 2-hour period on days 0, 5, 7, 10, and 15 of treatment before terminal blood sampling for basal TSH and T4 detection on animal euthanasia (Fig. 2C-D; males, black labels; females, red labels). All blood sampling were collected at the same time of the day for both male and female mice. Following 5 days of treatment, T4 levels were significantly decreased in both males (***P = .0006; 1-way ANOVA; n = 8 per group) and females (***P = .0002; 1-way ANOVA; n = 8 per group), whereas basal mTSH levels were unchanged. Starting from day 7, basal mTSH levels were significantly higher than at day 0 in both males and females (****P < .0001; mixed-effects analysis; n = 8 per group) and continued to increase during mouse LID treatment (Fig. 2C). Using the coefficient of variation parameter as an index of mTSH oscillatory patterns (Fig. 2D), our mTSH ELISA was sufficiently sensitive to show that the occurrence of ultradian acute TSH pulses progressively decreased starting from 7 days and 10 days of LID treatment in males and females, respectively (Fig. 2D-F). However, basal TSH began to increase (Fig. 2C), suggesting that the ratio between TSH pulses and basal TSH levels decreases in this mouse model of primary hypothyroidism, as previously reported in human hypothyroid patients (26).

### Ultradian mTSH Pulses are Highly Efficient at Stimulating the Thyroid

The previously unknown characterization of ultradian TSH pulses in mice led us to address a fundamental question regarding thyroid control that has not been possible to explore in human studies (4, 11, 12, 20); how does pulsatile vs sustained mTSH increases control downstream thyroid signalling and related circulating TH levels? Determining whether the thyroid gland has the capability to precisely sense and discriminate differences in TSH patterns is crucial to understanding their relevance.

**Figure 3.**
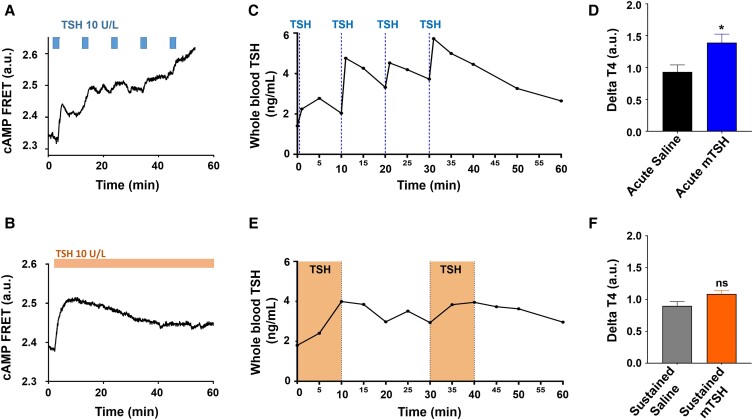
TSH pulse decoding by the thyroid. (A-B) Thyroid follicles isolated from CAG-Epac1-camps mice were visualized by time-lapse fluorescence microscopy (representative examples of follicle responses). FRET values are shown as normalized YFP/CFP ratio values. (A) Effect of pulsatile stimulation with 10 U/L of TSH on intracellular cAMP levels. (B) Effect of a continuous stimulation with 10 U/L of TSH on intracellular cAMP levels. Horizontal bars represent the time periods of TSH applications. (C-F) Anesthetized T3-treated mice received IV 8 ng TSH in either a pulsatile (C) or 16 ng perfused (D) pattern via the jugular vein. Administration of fast TSH pulses induced a significant rise in T4 3 hours after the beginning of TSH administration (E), whereas no significant increase in T4 was observed when mice received slowing evolving TSH rises or saline injection and perfusion (F). One sample Wilcoxon test *P = .0313. Data are shown as mean ± standard error of the mean. Results in (D-F) are average data of 5 to 10 experiments per condition.

We therefore designed 2 distinct protocols to test TSH pattern discrimination at the level of the thyroid gland. We explored whether the thyroid could discriminate TSH secretion patterns by applying either pulsatile or sustained TSH stimulation to isolated thyroid follicles from CAG-Epac1-camps mice (17). FRET monitoring of intracellular cAMP dynamics revealed that applying a series of TSH pulses that mimic the recurrence of endogenous ultradian pulses observed in freely behaving mice (Fig. 2) triggered a stepwise cAMP increase, whereas a sustained TSH elevation induced an initial rapid cAMP response, which then slowly declined despite the continuous TSH perfusion of thyroid follicles (Fig. 3A and B). These data suggest that the thyroid can efficiently discriminate between a series of repeated TSH pulses, which appear more effectively coupled to downstream cAMP signalling, and continues prolonged stimulation. This hypothesis was further supported by measurements of TH responses to distinct TSH patterns directly in vivo. Following initial treatment with T3 to blunt endogenous mTSH secretion (27), mice were anesthetized and a catheter was inserted in the jugular vein for blood collection and drug administration. Two mTSH administration protocols were used: first, mTSH was delivered at 10-minute intervals (Fig. 3C), thus modelling fast-rising mTSH secretory pulses observed in vivo (Fig. 2A); and second, sustained 10-minute mTSH perfusions that led to slowly evolving rises in basal TSH levels (Fig. 3D) were delivered. Blood was collected from the tail-tip every 1 to 5 minutes during intervention to longitudinally monitor mTSH administration. An additional 50-µL blood sample was collected from the jugular vein to measure circulating T4 before and 180 minutes following the first mTSH injection (27). Remarkably, a series of 4 acute mTSH pulses led to a significant increase in circulating T4 (Fig. 3E), which was not observed following slowly evolving increases in mTSH (Fig. 3F) 3 hours after the initiation of mTSH administration, suggesting a higher efficiency of recurrent, acute mTSH pulses on thyroid hormone secretion.

## Discussion

One of the major challenges in thyroid physiology and pathology is to identify the TSH levels that best regulate thyroid function (4, 26). Despite the wealth of information on TSH action in thyroid function, little is known about the role of ultradian TSH patterns in thyroid control, prompting us to develop a logical approach in mice to identify the characteristic features of ultradian TSH patterns and their relationship with thyroid functioning. Our experimental approach, which included a newly developed, highly sensitive sandwich ELISA for longitudinal assessment of mouse TSH microfractions of tail-tip whole blood, allowed us to decipher acute pulse patterns of TSH secretion in euthyroid freely moving mice; their changes in a mouse model of primary hypothyroidism; and their high efficiency at stimulating the thyroid, both at the level of cAMP production due to TSH receptor stimulation and T4 levels into bloodstream. This framework, which is readily applicable to a wide range of mouse models (28-32), provides one of the most generalized and precise ways to identify how TSH pulse patterns both occur and are functionally interpreted at the thyroid level in health and disease.

### Thyroid Sensing of TSH Secretion Patterns

Our approach has allowed us to address the long-standing question of downstream action at the thyroid level of circulating TSH profiles. Here, we revealed that the thyroid functions as a highly efficient “pulse detector” of ultradian TSH secretion patterns because sequential acute TSH pulses with a recurrence of “ON” and “OFF” episodes lead to step-sized increases in cAMP levels in thyroid follicles, whereas sustained TSH administration only leads to an initial cAMP response followed by a slowly evolving decay phase. This was further supported by T4 measurements following patterns of TSH administration that were simultaneously detected in individual animals.

If thyroid follicular cells can precisely decode TSH secretion dynamics, we would expect that alterations in the kinetics of signal transduction (cAMP) downstream of TSH receptor stimulation would lead to deleterious consequences for thyroid function. Using mouse models, it has been reported that TSH receptor activation in thyrocytes on a single, minutes-long TSH pulse triggers an initial cAMP response, followed by a second, more prolonged cAMP increase when TSH bound to its receptor is internalized and triggers intracellular Gs activation at the Golgi/trans-Golgi network (17, 33). This results in differential downstream effects such as rapid actin depolymerization, which is implicated in thyroglobulin reuptake and TH release and slow effects on gene transcription. Thyroid responses to circulating TSH pulses may therefore depend on thyrocyte reading of a series of acute TSH pulses, resulting in cAMP rises that may encode distinct thyrocyte functional outcomes. Recent studies have also revealed that increases in cAMP are shaped both in space and time by phosphodiesterases acting as local buffers (34, 35). Remarkably, pioneering genome-wide association studies in human patients initially showed an association of TSH levels with single-nucleotide polymorphisms in the phosphodiesterase *PDE8B* gene, which is highly expressed in the thyroid and is responsible for cAMP inactivation (36). Thereafter, there was an exponential discovery of genetic determinants of human thyroid dysfunction, with approximately 100 genes implicated in determining serum TSH levels, several of which regulate the Gs/cAMP pathway (*ADCY9, GNG7, GNG12, PDE4D, PDE8B,* and *PDE10A*) (37). We can therefore imagine that defects in spatiotemporal cAMP dynamics can lead to misinterpretation of thyroid encoding of ultradian acute TSH pulses in humans. Our experimental framework will now allow study of kinetically distinct TSH patterns and their outcomes in mouse models expressing targeted mutations of genes of this Gs/cAMP pathway at the thyroid level because signal-transduction processes can be conditionally altered in vivo in murine endocrine glands (eg, by using targeted CRISPR-Cas9 techniques) (38).

In summary, our results about the TSH patterns-thyroid function crosstalk in mice suggest a new way of thinking about the thyroid control by TSH secretion profiles. By using our newly developed, highly sensitive mTSH ELISA, it will now be possible to synergistically combine these longitudinal in vivo studies conducted over weeks to months in individual animals, which act as their own controls, with single-cell multi-omics studies (39) at key time points during the establishment of thyroid dysfunction. It is anticipated that the increased understanding this will provide will result in multiple follow-up studies to elucidate the clinical implications and the etiology of other thyroid defects (15, 16), and will allow identification of new predictive indices and refined guidelines, which are currently based on single point TSH detection, potentially leading to misdiagnosis and inappropriate levothyroxine treatment (15, 40-42).

## Data Availability

Original data generated and analyzed during this study are included in this article.

## Abbreviations

AUC: area under the curve
CFP: cyan fluorescent protein
CV: coefficient of variation
FRET: forster resonance energy transfer
LID=LoD: low-iodine diet
mTSH: mouse thyroid-stimulating hormone
RT: room temperature
T3: triiodothyronine
T4: thyroxine
TH: thyroid hormone
TRH: thyrotropin-releasing hormone
YFP: yellow fluorescent protein
